# Can a novel set of handgrips on a walking frame increase stability and support users when transferring to/from a chair?

**DOI:** 10.1186/s12877-025-05754-7

**Published:** 2025-02-20

**Authors:** Sibylle Brunhilde Thies, Susan Bevan, Matthew Wassall, Cynthia Poolay Mootien, Laurence Kenney, David Howard

**Affiliations:** 1 Centre for Human Movement and Rehabilitation, School of Health & Society, University of Salford, Brian Blatchford Building Room PO28, Salford, Greater Manchester UK; 2 NRS Healthcare, Coalville, Leicestershire LE67 1UB UK; 3 School of Science, Engineering and Environment, University of Salford, Salford, Greater Manchester UK

**Keywords:** Walking frame, Stability, Usability, Design, Sit to stand, Transfer

## Abstract

**Introduction:**

One important aspect of walking aid use is transferring safely to the aid from sitting and transferring back to the chair after walking, since these activities have been associated with falls in older adults. Standard frames require their user to push off the chair or ask for help from a carer, which may over time lead to back pain. This study’s aim was to assess whether novel handgrips located above the rear feet of a walking frame would facilitate safe transfer as compared to utilizing only the seat cushion or armrests of the chair.

**Methods:**

In a gait lab-based trial 10 healthy older adults repeatedly transferred from sitting to standing, pushing off the chair’s seat cushion, armrests, or using the new lower handles on the frame (alone or in combination with the seat cushion or armrest). The stability margin ‘SM’, defined as the distance between the centre of pressure and the nearest edge of the base of support for the user-device-chair system, was calculated as a mechanical measure of stability. Specifically, SM provides a measure of how close the system is to the point of tipping over. Additionally, 13 older frame users tried to use the new handgrips to transfer to/from the new frame and gave interviews which were thematically analysed.

**Results:**

Stability for the 10 healthy older adults was statistically either equivalent or better when using one or both handles on the novel frame as compared to pushing off the chair’s seat cushion or armrests. Amongst the 13 older frame users the frame’s new handgrips were useful to those living in the community and one person living in care, and they perceived them to facilitate independence and control.

**Discussion & conclusions:**

The novel handgrips offer continuous support when getting up/sitting down and are well-received by those able to use them. The significance of the research lies in the reported number of falls during transfer from sitting to standing and vice versus, with underlying causes reported including loss of support. The proposed design is timely considering the documented increases in frailty and walking aid use in our ageing population.

## Introduction

Due to continued population ageing [1], falls in older adults remain an ever-growing health problem [2, 3] which costs the UK £2.3 bn annually [4]. Walking aids are a common intervention for those with mobility issues, as they have been designed to enhance mobility and stability and thereby prevent falls, and they are used indoors alone by approximately 22% of older adults living in the UK (and 48% of older adults use an aid outside) [5]. Nevertheless, a perplexing association between walking aid use and falls [6–9] as well as fear of falling [10] has been reported, and hence a number of studies have investigated static stability of the device alone in accordance to ISO standards [11] as well as the biomechanical stability of walking aid users [12–17]. However, the majority of these studies assessed stability during walking and/or turning, whilst transferring to the walking aid from sitting, and sitting back down from standing, are two activities that remain relatively unexplored. It has been shown that transferring up/down from sitting to standing and vice versa is associated with falls in frail older adults [18]. If a walking aid user is not able to safely transfer to/from their device their mobility will remain limited by the need for assistance, which may in time impact on back health of those that provide support [19].

Interestingly, one recent study involving healthy individuals found that utilizing support from the handles of a simulated, rollator-like frame during sit-to-stand and stand-to-sit manoeuvres facilitated a stable transfer [20]. These findings are encouraging and show the potential for assistive devices to support transfer activities. However, the frame used in their study was designed such that it could not tip over, whilst a normal rollator would be prone to tipping if a user attempted to lift themselves up pulling on the handles which are typically located at the top of the device and far forward from the body and the frame’s rear feet.

In a Knowledge Transfer Partnership, we recently designed a novel walking frame informed by biomechanical analyses of stability and user and clinician feedback, to overcome some of the shortfalls we had previously observed with standard frames [16]. The new design introduced larger-diameter swivel wheels at the front that are subject to a magnetic forward bias, glider feet at the rear, and additional handles to support getting up/sitting down [21]. We have already demonstrated the benefits of this novel frame on walking and turning activities [21], using the stability margin for the combined user-frame system as a mechanical measure of stability [14]. Specifically, we defined the combined stability margin ‘SM’ of the user-frame system as the distance between the system’s centre of pressure ‘CoP’ and the nearest edge of the system’s base of support ‘BoS’. In general terms, SM provides a measure of “how close the user-device system is to the point of tipping over” [21]. This study is novel in that we have extended our approach to stability computation to include the chair as part of the system whenever the user is in contact with the chair during getting up/sitting down activities, i.e. at a given moment in time the stability margin is computed either for the person alone, the person and their frame, the person and the chair, or the person, their frame and the chair—depending on what object(s) the person is in contact with at that time. Here we are using this method for the first time with the primary aim of assessing whether the use of the novel handgrips, located at a lower level and above the rear feet of the new prototype frame, would increase stability when transferring from/to a chair without assistance from another person, compared with performing the same tasks following the standard clinical guidance to push off the chair. The secondary aim of this study was to explore users’ perceptions regarding getting up and sitting down supported by the new prototype frame’s handgrips versus transferring with support from the chair as per guidance.

## Methods

Institutional ethical approval was obtained for both the qualitative and quantitative work (Ref#2669).

### The standard and the novel prototype walking frames

**Fig. 1:**
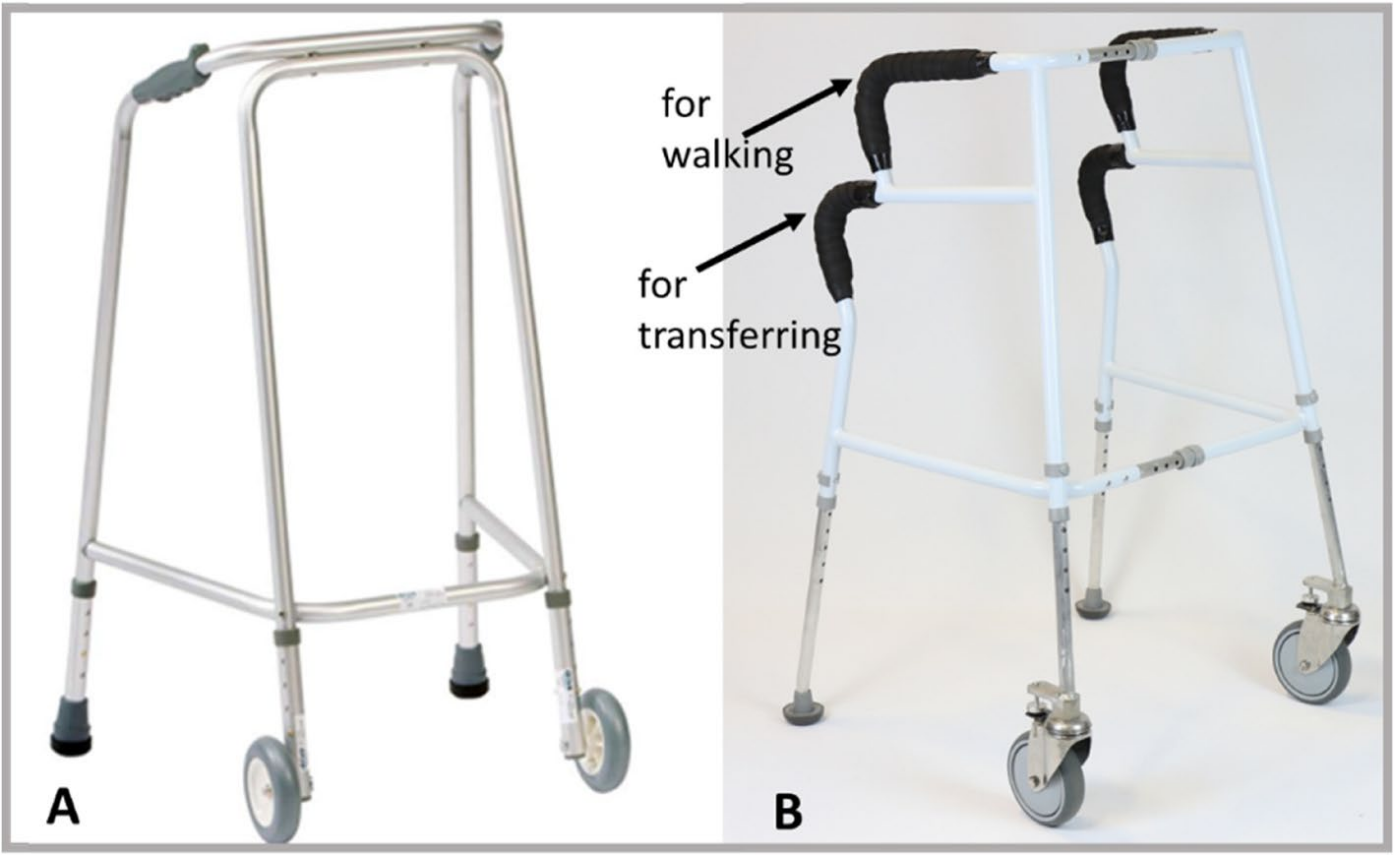
Standard frame (**A**) and prototype frame (**B**) adapted from [21]. The prototype frame also featured brakes inside the rear feet, reducing risk of it running away from the user

Front-wheeled indoor walking frames, hereafter referred to as standard frames, have one set of handgrips at the top where the user places their hands during walking. Because of their location (in front of the rear feet of the frame and high up) these handgrips are not to be used to rise up from a chair or when transferring to sit down, as the frame may tip towards the user. Instead, clinical guidance recommends that the user pushes off the seat or the armrests of a chair. The new prototype frame shown in Fig. 1 features an additional set of hand grips at the rear, and users may use both of these handgrips or may have one hand on one grip and the other on the seat or armrest of the chair when rising up or sitting down. Importantly, when pushing down on these new handgrips, an inner braking element on the inside of the frame’s foot makes contact with the ground, and through friction this reduces the risk of slipping of the prototype frame, making it a stable object to push up from or lower down from. Notably, the novel handgrips differ in that they were made of memory foam wrapped in textured banding for greater comfort and reduced pressure on the hand, whilst the grips of standard frames are made of hard plastic.

### Quantitative study

#### Participant recruitment

Ten healthy older adults without mobility issues were recruited for the gait lab-based study (a group of 6 males & 4 females, age: group mean ± SD = 61.3 ± 4.3 years. Weight males: group mean ± SD = 81.7 ± 7.2 kg, weight females: group mean ± SD = 63.8 ± 12.9 kg). Exclusion criteria were: 1) a history of head injury or concussion, 2) visual disorders not correctable by glasses, 3) in self-isolation/quarantine due to COVID. These adults were recruited from the staff population at the University of Salford. Only healthy participants were included because actual users of walking frames would not have been able to perform the required protocol (rising up from a chair and sitting down again, 15 times each) due to their intrinsic frailty; 4–6 repeats had already proven challenging with actual walking frame users in our previous work [21]. All received a Participant Information Leaflet and provided informed consent.

#### Data collection

For this study, two tasks were assessed: a) standing up from a chair as to get ready for walking with the walking frame, and b) sitting down on a chair (from standing in front of the chair with the frame, i.e. as if walking with the frame had just finished). The height of either frame was set so that the top handles were at the level of the wrist when standing with the frame, i.e. as per clinical guidance.

**Fig. 2:**
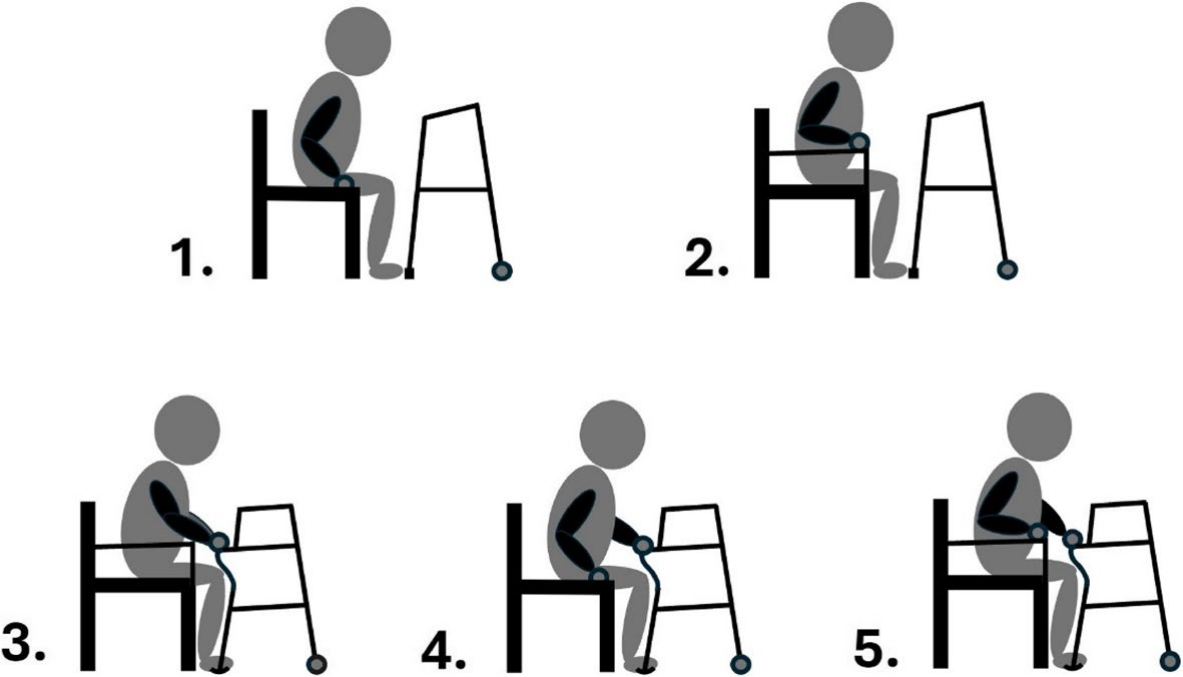
Visualizations of the 5 conditions to transfer from the chair to the walking frame and back down to the chair

For these two tasks “getting up” and “sitting down”, 5 conditions (shown in Fig. 2, with 3 repeats per condition) were performed by each subject:

1. Standard frame, pushing off the chair’s seat cushion with both hands.
2. Standard frame, pushing off both armrests of the chair.
3. Prototype frame, pushing off both new lower handgrips of the frame.
4. Prototype frame, one hand pushing off one of the new lower handgrips of the frame and the other hand pushing off the chair’s seat (left or right hand as preferred).
5. Prototype frame, one hand pushing off one of the new lower handgrips of the frame and the other hand pushing off the chair’s armrest (left or right hand as preferred).

Data collection took place in a gait laboratory at the University of Salford and was managed by the authors (SBT, SB, MW). Each participant volunteered on a single occasion for approximately 1 h including setting them up with instrumentation, data collection during task performance, and breaks. As in our previous work [14–17, 21], we collected data using 3D motion analysis (Qualisys Oqus300, Qualisys AB, Göteborg, Sweden) to obtain position data of the user’s feet by tracking reflective markers placed on both shoes (at the approximate location of the 1st, 2nd and 5th metatarsal head and the calcaneus), the walking frame’s feet and wheels, and the chair’s feet. Marker position data were recorded in conjunction with data from load cells (Futek LCM300, Futek Advanced Sensor Technology Inc., Irvine, CA, USA) fixated inside the walking frame’s legs, and data from pressure-sensing insoles (medilogic®insole, T&T medilogic Medizintechnik GmbH, Schönefeld, Germany) inside the user’s shoes. In addition, two Advanced Mechanical Technology, Inc. ‘AMTI’ force plates recorded the chair’s weight plus any body weight placed on the chair (AMTI Force and Motion, Watertown, MA, USA). All data were collected at 100Hz and the different measurement systems were temporally aligned through use of a sync pulse [14].

#### Data analysis

Notably, in our previous stability analyses of walking with a walking frame we treated the user and their walking frame as a single moving system for as long as the user was holding on to their walking frame [14–17, 21]. Our outcome measure, the “combined stability margin” is computed from the forces of those feet in contact with the ground (anatomical feet and/or walking aid feet) together with their position relative to each other (providing centre of pressure and base of support). For this study, we extended our custom-programmed Matlab® algorithms and added the chair into the system at times where the user was still in contact with the chair, with contact defined by a 2N threshold exceeding the chair’s weight, as determined by the AMTI force plates. The instrumentation outlined above provided the data required to define the user-device-chair system’s combined centre of pressure, combined base of support, and the associated combined stability margin. Specifically, stability margin values for the user-device-chair system were calculated during performance of the tasks as described in [14], with the addition of the chair being at times part of the system. Hence stability at a given time was computed for the user alone if they did not contact the chair or frame, or for user and chair, or user and frame, or user and chair and frame combined – i.e. any object the user was in contact with was considered part of the system at that time. The combined stability margin of the system over time then reflects how close the system is from the point of tipping over.

From the stability margin trajectories, two outcomes were obtained (for each of the 5 conditions within each task, i.e. within ‘standing up’ and ‘sitting down’):

- *The average SMmin (‘Avg SMmin’):* for each of the 3 repeats of standing up/sitting down, the minimum stability margin value was computed, and then the average was taken across the 3 repeats.
- *The minimum SMmin (‘SMmin’):* across the 3 repeats of standing up/sitting down, the minimum stability margin value was obtained, reflecting the instant where the system was closest to the point of “tipping over”.

#### Statistics

The above defined outcomes ‘Avg SMmin’ and ‘SMmin’ data for all 5 conditions were checked for normality. The Friedman’s test, a nonparametric test for data that is not normally distributed (equivalent to the repeated measures ANOVA which is only to be used on normally distributed data) was then used. The Friedman test identifies whether there are statistically significant differences between 3 or more dependent samples by assessing for a difference in rank totals (rather than means) between the conditions. It was first used to test for a difference in ‘Avg SMmin’ between the 5 conditions for ‘getting up’, and, secondly, was then used for testing for a difference in Avg SMmin between the 5 conditions for ‘sitting down’. The same was then repeated for ‘SMmin’ ‘getting up’ as well as ‘sitting down’. Pairwise comparisons then informed on where amongst the 5 conditions the difference lies. These post hoc test were conducted with a Bonferroni correction applied, i.e. to account for multiple comparisons, significance values were adjusted by the Bonferroni correction for multiple tests. This reduced the chance of making a Type I error: erroneously rejecting the null hypothesis when it is, in fact, true.

### Qualitative study

#### Participant recruitment

Thirteen participants who used wheeled walking frames and were able to walk household distances with their frame were recruited from the community through advertising at relevant organizations/locations (e.g. community cafes), social media, and word-by-mouth. Seven were care home-based residents (3 females, 4 males, age: mean ± SD = 88.71 ± 6.92 years) whilst 6 were community-living (5 females, 1 male, group age: mean ± SD = 78.17 ± 11.77 years, one house-bound and 5 community ambulating). Exclusion criteria were: 1) a history of head injury or concussion, 2) visual disorders not correctable by glasses, 3) in self-isolation/ quarantine due to COVID or symptoms of COVID. All provided informed consent.

#### Data collection

The specific tasks performed by participants were as follows:

1. Getting up with their own frame, pushing up from the seated surface, followed by reaching for the seated surface for support when sitting down.
2. Getting up with their own frame, pushing up from the armrest of the chair, followed by reaching for the armrest for support when sitting down.
3. Getting up with the prototype frame, both hands on the left and right lower hand grips. Followed by the same process to sit back down.
4. Getting up with the prototype frame, one hand pushing from the chair (seat or armrest as preferred) and the other hand on the additional, lower hand grip of the frame. Followed by the same process to sit back down.

Data collection took place either in the participant’s home or a place of their choice near their home, e.g. community room/café, and was managed by the authors (SBT, CPM). Each task was only performed once and only if the participant felt safe and able to attempt a given task. Participants were offered the opportunity to take as long a break as they needed between tasks. Following the task performance, participants were then interviewed face-to-face by the author CPM regarding their experience using the prototype frame and utilizing its lower handgrips for support when getting up and sitting down. An interview guide containing predetermined semi-structured interview questions, was used to circumvent errors that could arise during the interview, such as inconsistencies in the questioning process, unnecessary probing, or bias. The semi-structured interviews [22, 23] lasted 10–15 min and were audio recorded and included questions such as:

- What do you think of this new walking frame compared to the standard wheeled frame?
- What did you think of the lower hand grips on this frame?

Follow-up questions were also asked, such as:

- How does this affect you?
- How do you feel about this?

#### Data analysis

Data triangulation was carried out to get a more comprehensive understanding of participants’ views and experiences of using the new frame, whereby the data from the 2 qualitative phases of data collection was anaylsed. This included observations of participants using the new frame while specific tasks were performed, and audio-recorded interviews with participants providing detailed exploration of their opinions and experiences of using the new frame.

All interviews were transcribed verbatim, after which thematic analysis was used for data analysis, so that patterns could be systematically identified and interpreted to better understand experiences and views across the qualitative data set [24, 25]. The analysis involved 6 stages, namely familiarisation, coding, developing themes, reviewing themes, defining themes, and reporting. Transcripts were analysed and coded by author CPM, to identify the initial themes. Microsoft Excel was used to collate and categorise the themes identified. The overarching themes emerged from the investigation of the codes generated from thematic analysis. Once these themes had been drawn from the data set, discussions were held with authors CPM and SBT until consensus was reached on the final overarching themes to be included in the study.

## Results

### Quantitative study

**Fig. 3:**
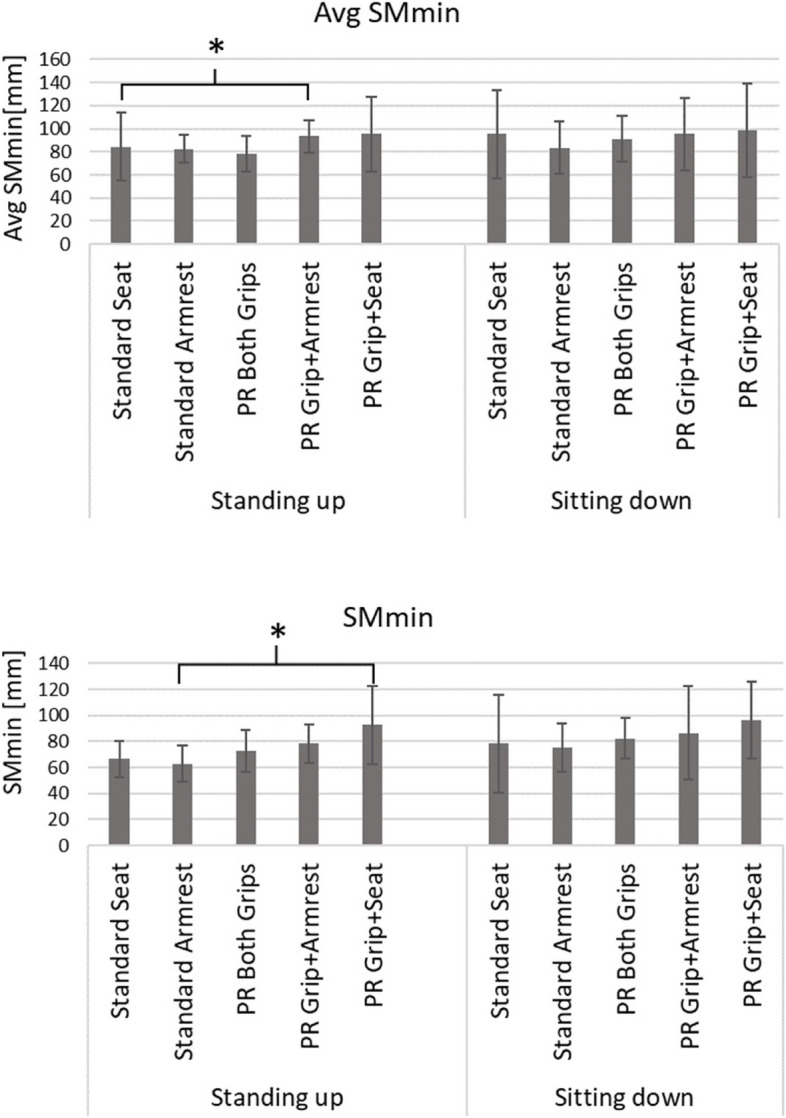
Stability results for the various test conditions of “Standing up” and “Sitting down”, using the standard frame “Standard” or the prototype frame “PR”, with the following hand positions: both hands on seat “Seat”, both hands on armrest “Armrest”, both hands on grips of the prototype frame “Both Grips”, one hand on grip of the prototype frame and the other on the armrest “Grip + Armrest”, and one hand on grip of prototype frame with the other on the seat “Grip + Seat”. Shown are group averages and standard deviations for the average SMmin (‘Avg SMmin’, top) and the minimum SMmin (‘SMmin’, bottom). *P* values of < 0.05 for paired comparisons are indicated with “*”

Figure 3 shows group averages and standard deviations for the average SMmin and minimum SMmin values. For standing up from sitting, comparison of the average SMmin and minimum SMmin values across conditions respectively resulted in Friedman test *p*-values of 0.011 and 0.007, respectively, leading to the rejection of the null hypothesis that there is no difference in respective stability margin value across the 5 conditions. Specifically, looking at the underlying pairwise comparison for average SMmin values this was due only to the comparison *“Standard frame, pushing off chair’s seat cushion with both hands (mean* ± *SD* = *84.55* ± *29.59mm)”* with *“Prototype frame, one hand pushing off the new frame grip and one off the chair’s armrest (mean* ± *SD* = *93.68* ± *13.91.59mm)”* (*p* = 0.029), whilst looking at the underlying pairwise comparison for the minimum SMmin values this was due only to the comparison *“Standard frame, pushing off both armrests of the chair (mean* ± *SD* = *62.90* ± *13.97mm)”* with *“Prototype frame, one hand pushing off one of the new handgrips and the other hand pushing off the chair’s seat (mean* ± *SD* = *92.65* ± *30.18mm)”* (*p* = 0.006).

However, comparison of the average SMmin values observed for sitting down from standing, resulted in a *p*-value of 0.825. Likewise, comparison of the minimum SMmin values observed for sitting down from standing, resulted in a *p*-value of 0.631.

### Qualitative study

Of the 13 wheeled walker users only six participants used the novel lower handgrips for support when getting up and sitting down (the 5 community-based ambulating participants and 1 care home resident). Nevertheless, all were interviewed to explore their views on the new lower handgrips. Table 1 shows descriptive information for participants in relation to their mobility and ability to get up and sit down in various ways.

**Table 1:** Participants’ demographic and mobility characteristics

Participant ID	Gender	Age	Walking frame experience	Mobility
1	Female	75	3-wheeled rollator, tea trolly, front-wheeled Zimmer frame.	Gets up very easily in general, pushing off the chair’s seat, armrest or novel handgrips.
2	Female	75	4-wheeled rollator, front-wheeled Zimmer frame.	Gets up very easily in general, pushing off the chair’s seat, armrest or novel handgrips.
3	Female	85	3-wheeled delta rollator, front-wheeled Zimmer frame.	Independent walking and getting up but struggled with repeatedly rising up from chair – showing some frailty/fatigue but able to get up pushing off the chair’s seat, armrests or novel handgrips.
4	Female	75	4-wheeled rollator, front-wheeled Zimmer frame.	Gets up very easily in general, pushing off the chair’s seat, armrest or novel handgrips.
5	Male	62	4-wheeled rollator, front-wheeled Zimmer frame.	Struggles to get up in general, usually cannot get up without support or assistance, but was able to utilize both lower handgrips on the novel frame to get up.
6	Female	97	4-wheeled rollator, front-wheeled Zimmer frame.	Tried but was not able to perform the tasks due to pain, frailty, and fatigue. Could not get up in general without an inclining chair.
7	Male	92	Front-wheeled Zimmer frame.	Tried but was not able to get up using the new lower handgrips. Was not able to get up without help in general.
8	Male	95	Front-wheeled Zimmer frame.	Usually struggles with getting up in general. But was able to get up using one of the new lower handgrips and pushing off the chair’s armrest with the other hand.
9	Male	75	Front-wheeled Zimmer frame.	Tried but was not able to get up using the new lower handgrips. Was not able to get up without help in general.
10	Female	92	Front-wheeled Zimmer frame.	Tried but was not able to get up using the new lower handgrips. Was not able to get up without help in general.
11	Female	92	Front-wheeled Zimmer frame.	Tried but was not able to get up using the new lower handgrips. Was not able to get up without help in general.
12	Female	91	Front-wheeled Zimmer frame.	Tried but was not able to get up using the new lower handgrips. Was not able to get up without help in general.
13	Male	84	Front-wheeled Zimmer frame.	Tried but was not able to get up using the new lower handgrips. Was not able to get up without help in general.

The overarching themes that were identified during thematic analysis were *‘Apprehension trying a new frame’, ‘Independence and sense of security’, ‘Being in control’, *and *‘Acceptance of prototype frame as being an improvement on the standard frame’.*

#### Apprehension trying a new frame

Six participants were confident when using the new prototype frame to get up and sit down without fear of falling or losing control, for example:

“Good idea that is to have [the lower handgrips]. Helps you so, your shoulders and everything else. Good. It co-ordinates” (P8)

However, apprehension before and while trying the new prototype frame was observed with those interviewees who had had negative experiences with standard walking frames. These participants were particularly vocal about not liking standard frames, describing the frames as being unsteady, hence making them feel unsafe:

“I’ve only used the standard one when I’ve had operations in hospital… That felt very flimsy… I had a hip, or a knee done, so not very secure on my feet, and I didn’t really like that [Zimmer] frame. It didn’t feel very safe, whereas I think I would have liked that one [the prototype frame].” (P2)

This sense of not having proper control added to the sense of insecurity and fear of using frames, in part out of fear of falling, with some being more worried about the standard frame and others remaining worried about the prototype frame:

“Well, I don’t think the ordinary Zimmer is very safe to be honest. Because they don’t have any brake system […] Zimmers can just roll away, you know… That [the prototype frame] seems to be…seems to be more sturdy than an ordinary Zimmer as well.” (P4)

“I felt that it [the prototype frame] could run away more easily from me […] In other words, I could fall more easily than with that one [the standard frame].” (P13)

Even after they had been shown where the brakes were on the prototype frame and how they worked, some remained worried about it running away when using it to get up/sit down.

However, most participants who displayed apprehension about the prototype frame also agreed that its novel design made it a good and practical frame to use, but commented that they had used theirs for a long time, and mentioned that using the prototype frame to get up and sit down would be challenging for them.

“I think it is a good idea, what you have done, yeah. But I found it difficult because it was new.” (P12)

“Though I may get used to it [referring to the prototype frame].” (P10)

#### Independence and sense of security

Several participants were very vocal about their approval of the addition of the lower handgrips on the new frame, with independence when rising from a chair being viewed as important to these users as having a frame that provides good mobility. For example, P2 explained that struggling to get up from a seated position and having to ask for assistance did not allow them to be or feel independent. This user went on to state that not only did they feel secure with the prototype frame, but using the lower handgrips on the prototype frame was better than asking for assistance when rising from a chair.

“…somebody has to come and give you a lift. It sort of spoils it because it takes away your independence […] No, it felt very, very, secure. It felt better than somebody helping me up.” (P2)

P4 explained that the lower handgrips were a good addition to the prototype frame, as the height of these handgrips allowed shorter people to exert enough pressure on the handgrips, to get up from a seated position without struggle or needing assistance. They also found the higher handgrips on standard frames more challenging to use when getting up from a seated position (in fact they are not meant to be used for getting up and sitting down but users do so nevertheless):

“They [the new lower handgrips] were a good addition for me because they’re exactly […] the right height for me, you know what I mean, to put pressure on…if they had been a bit higher up it would have been more difficult to put pressure on to stand up […] if you’re quite tall it’s easier, but you’re small and you’ve got short arms, you know… it seemed to be absolutely perfect [the lower grips] the right place for me and my height […] so yeah it felt good.” (P4)

Moreover, P5 pointed out that the lower hand grips were of particular interest to users who would normally struggle to get up from soft seating, such as couches.

“They’re [lower grips] much better if you are sitting on a cushioned base […] you’ve got the choice because you’ve got the two levels and you would choose the level you need for those two things [transferring to/from the frame and walking].” (P5)

Participants further stated that the soft handgrips on the prototype frame felt safer to use than those on standard frames, providing a sense of security:

“No, no you feel secure. Like some handles even they’re just ordinary painted ones, your hand does slip down them.” (P3)

“It felt quite secure, it felt like […] my hand wasn’t going to move on it. That’s what happened, my hand didn’t move.” (P4)

“I think the rubber specifically gives you some sort of confidence […] and the other thing is you can actually squeeze them, and when you squeeze them, you can feel that you’re getting a grip on them.” (P5)

“Yes, and the stuff that’s on it, it grips… Because if I’ve got an ordinary piece of metal, you know, like the white part [indicating handgrips on their own frame], it tends to slip.” (P6)

“I liked these. I like to have a little bit of sponge in them. So, if I got a bit tense it gives me a little bit more strength and sturdiness if that makes sense”. (P9)

Regarding the fixation of the lower handgrips, participants appreciated that the angled handgrips prevented awkward hand manoeuvres whilst helping them push off from a sitting to a standing position using the prototype frame. P3 explained that the fixation of the lower handgrips on the prototype frame was an improvement to the handgrips on standard frames, which allowed better control of hand and arm movement when using the frame to get up (although the standard frame is not meant to be used for this as mentioned above).

“Yes, I think that is an improvement on the ordinary handle position […] rather than up here where you lose a lot to the strength from your arms […] I think that is the right position for them, rather than straight at you. I think that gives you the push up easier because you’re on an angle. I think that is better.” (P3)

This was also reiterated by P2:

“I thought it was a brilliant idea because you can already get a good grip to start pushing yourself up, and then walk up […] I thought they were in a very good position to be honest.” (P2)

Almost all users found that the design of the lower handgrips was novel and practical, particularly with regards to having the choice of two sets of handgrips for different situations. They also liked the softness and comfort provided by the material on those handgrips, as well as the fixation of the lower handgrips. These features allowed some users to feel independent as well as safe and secure.

#### Being in control

Being in control of the walking frame was a recurrent theme that emerged throughout the interviews. This theme was also noted when users discussed what they liked or disliked about the prototype frame, and what they preferred with their own frames compared to the prototype frame. There was consensus among all participants that the dimensions of the prototype frame were different to that of standard frames, with some users finding it heavier or bulkier; participants’ views on whether this was a positive or negative aspect of the prototype frame varied. However, the theme of being in control emerged from all discussions about whether users liked or dislike the weight and dimensions of the prototype frame. Therefore, ‘being in control’ had to be reported as an overarching theme as it was linked to two main themes, namely ‘dimensions and security’ and ‘bulkiness and impracticality’.

##### Dimensions and security

Many participants felt that the new frame was heavier than the standard one, which was due to the larger wheels designed to allow for better mobility. P2 admitted that walking frames would have to be heavier in order to improve on their sturdiness and stability, implying that it was impossible to have both a sturdier and lighter frame. They also admitted to finding all walkers bulky, but that this is acceptable when stability is one of the most important aspects needed in a walker.

“I didn’t not dislike anything about it, other than it’s a little bit bulky. But then they all are, aren’t they? But I don’t think you can do anything about that because you need that stability.” (P2)

It was also observed that users would not fall so easily with the new frame as it was studier compared to other frames:

“It’s more solid [the new frame] yes, and if you were going to fall, which I did do with that one [their standard frame], you wouldn’t fall so easily with this [prototype frame] […] It felt more secure shall I say. That’s [the standard frame] a bit…they are more flimsy, aren’t they?” (P10)

“It’s much better, it’s much more sturdy, and you feel more relaxed and safe with it really…” (P5)

##### Bulkiness and impracticality

Interestingly, even though some participants agreed that the weight of the prototype frame added to its sturdiness and hence helped users feel safer while using it, they also pointed out the impracticalities of having such a frame. For example, P1 felt that the prototype frame was safe, and agreed that the reason for this was due to the frame’s sturdiness, which would prevent it from tipping easily.

“Oh, I think it’s safe. I think it’s safe because it’s sturdy, isn’t it? You know, I don’t think that could tip.” (P1)

However, this participant also described their issues with the weight of the new frame:

“I didn’t like it [the weight]; it was heavy and to position it’s going to be awkward because you have to drag and pull to get yourself in position. I suppose if you got used to it, you’d know exactly which position, but I didn’t feel comfortable with it.” (P1)

This participant did not feel in control while using the frame to get up and sit down, as it was perceived to be difficult to position and manipulate for these manoeuvres.

Similarly, P3, who had felt safe and secure when using the new frame due to its stability and sturdiness, nevertheless also found it too sturdy and explained that weight was an important factor that they had considered when buying their walking frame, which is lighter than the new frame:

“Yes, probably a bit too sturdy really for moving around in the house […] I didn’t like the bulkiness of it, the weight of it. You know, if you had to lift it up a step or something. That’s the beauty of my light one […] I bought it specifically because it was light and could be put easily into somebody’s car boot without them struggling too much.” (P3)

One user also mentioned that the weight of the new frame would become more problematic over time, implying that as the user gets older, frailer, and potentially more ill, the frame would be impractical for them to use.

“I think it’s quite good. I mean I think I would get used to it very quickly […] But I suspect, the weight of it is going to get more of a problem to me.” (P11)

In summary, dimensions and associated bulkiness affected participants’ perceptions of being in control, for better (in terms of sturdiness reducing the risk of falling) or worse (in terms of being able to manoeuvre it). The authors note that the frame did carry instrumentation (4 load cell-transmitter pairs, a total of 1 kg) at the time of the study, the weight of which may have exaggerated these findings.

#### Acceptance of new frame as being an improvement on the standard frame

Although there was debate as to the practicality of the prototype frame in home settings and for transporting, participants did find the novel aspects of the prototype frame had their benefits and that the prototype frame was an improvement on standard frames:

“I liked the grip on the handlebars, I like the 2 tiers on the handlebars […] and I sort of thought why has nobody thought of this before?”

Other participants agreed that adding the lower handgrips to standard frames, such as the Zimmer frame “would be a good idea really” (P5), and P12 stated:

“I thought that was a brilliant idea on the handles.” (P12)

However, it is important to note that not all users found the prototype frame’s lower handgrips enabling. Although the addition of the lower handgrips were perceived as practical and a major improvement on the standard walking frame for some, these changes appeared not to be beneficial to all users, particularly those who were frailer such as the care home residents with limited mobility and independence. The lower handles of the prototype frame therefore particularly benefited more mobile users.

## Discussion

This is the first study that assessed rising out of a chair and sitting down in the context of use of a standard frame (where the recommendation was followed to use the chair for support) versus use of novel handgrips located above the rear feet of a new prototype frame. The approach is novel, in that it considers not only the walking frame but also the chair in combination with the person for stability calculation, depending on what object(s) the person is in contact with at a given moment in time. Findings from the quantitative stability assessments were further substantiated through qualitative interviews that explored how frame users perceived the lower handgrips to help with transferring up from the chair to the frame and vice versa.

*In the quantitative work,* for the standard frame, clinical and manufacturers’ instructions were followed. The standard frame was not used during these two tasks; instead users were instructed to push off the chair’s seat cushion or armrests when standing up/sitting down. The prototype frame, however, had been designed to support users in these tasks directly, either by letting the user push off both lower handgrips or by using only one of the handgrips in combination with the chair’s seat cushion or armrest. Notably, the quantitative study on healthy older adults revealed that across test conditions stability was either equal or better when using one or both handles of the prototype frame as compared to just pushing off the chair’s seat or armrests. That the stability margin did not decrease for the new design is a vital outcome considering that previous work had linked a reduction in stability to falls and a history of falling [26, 27], and the data support the idea that the new frame can safely support standing up and sitting down if used for these transfers.

One limitation of the quantitative work lies in the small sample size which limits generalizability and interpretation of the statistical results. Another limitation was that the stability data were obtained for healthy older adults only; only healthy were included because frail frame users would not have been able to do the required number of repeats in this part of the study (15 times rising up from sitting, and 15 times sitting down from standing). In our previous work we were only able to record 4–6 walking trials with actual users which naturally included getting up and sitting down [21], hence we chose healthy adults for this work that would have more than doubled what they had already found challenging in the past. Nevertheless, the findings from this study suggest that the new lower handgrips of the prototype frame safely supported these healthy users when transferring from sitting to standing or vice versa, something that current frames are not designed to do. To substantiate these findings, a longitudinal study of safety during transfer in older frame users is needed.

*In the qualitative work,* 6 out of 13 actual frame users were able to use the new lower handgrips to get up from a chair and sit down again, and those were mostly participants who were community-living (except for one care home resident). Participants were positive regarding the increased independence that the new handgrips would bring for them, reducing their need to ask for assistance. Notably, the seven participants who were not able to use the handgrips to get up/sit down were in general not able to get up from a chair without assistance, and the handgrips did not change that. Hence it seems that their usefulness depends on the degree of assistance required. Moreover, the interviews revealed that not being used to the new frame and concerns about it rolling away when pushing down on it (despite the invisible brakes, and based on their bad experiences with Zimmer frames rolling away) may have contributed to participants not using the frame’s lower handgrips for support when getting up/sitting down.

Last but not least, comments made regarding the extra weight of the prototype frame revealed an interesting trade-off: on the one hand users were concerned about pulling the heavier frame towards them into position when needed, or getting it into a car, yet they also perceived the extra weight as a positive as it made the frame more stable and secure.

We are aware of only one other study that assessed stability for standing up and sitting down with the support of the handles of a simulated rollator frame [20]. That work also identified that the handles facilitated a stable transfer to and from the frame. One key difference, however, was that their simulated frame did not tip due to its heavy weight. It has been our observation that use of the top handles on any walking frame can lead to backwards tipping of the frame, and is clinically nor recommended. This may, at least in part, be due to the handles being high up and forward. The novel frame in this study has its handles at a lower level, specifically at a similar level to the armrests of the chair, and right above the frame’s rear feet, i.e. closer to the user’s body. If a push force is applied to these handles the moment about the rear feet is zero due to a zero moment arm and the frame hence does not tip. Moreover, the push force activates “brakes” inside the rear feet that reduce the risk of the frame running away from the user. Notably, for the 6 users who used the new handles to get up and sit down we did not observe any sliding or tipping of the frame; the frame remained solid in its position in front of the chair. Whilst the new frame represents overall a novel design that differs from rollators and Zimmer frames in a number of ways, we believe that the new handgrips could also be integrated into existing frame types to enhance function.

It must be noted that some users found it difficult to adjust to the prototype frame as they were used to getting up and sitting down as per clinical guidance (pushing off the chair) and found it difficult to change their ways. This, however, may be addressed through appropriate guidance videos and leaflets since previous work has shown that training of users can facilitate effective use of mobility aids [28]. Yet the issue of adjusting to a new usage pattern may remain in those older adults that have cognitive deficits and who may struggle with retaining the information as to which set of handgrips is for what activity (transferring versus walking). Hence work is yet needed to trial the new handgrips’ suitability for use by older adults with cognitive impairments such as dementia and who already are used to rise up/sit down using the seat’s surface/armrest. We also acknowledge that whilst the overall frame height was adjusted following standard clinical procedure so that the top bar was at the wrist level during standing, this may have impacted the effectiveness of the lower handles in supporting sit-to-stand maneuovres, i.e. the taller the frame height setting, the higher the lower handles will be and this may make them more difficult to use. Independent adjustment of the lower handles’ height may be beneficial.

Notably, we utilized a mixed method approach, which has also proved useful in the evaluations of an intelligent mobility aid [29]. Similarly, our novel frame may also in future benefit from intelligent sensing capacity to guide the user to a safe standing/sitting posture via feedback on body weight support and posture. Indeed, recently similar advances have been made for an outdoor rollator [30]. However, further work is needed to understand the implications of such a technological enhancement, which would come with the burden of a need to charge regularly and research would be needed to understand ways to safely convey feedback to users.

*In conclusion*, the gait lab-based stability assessment of healthy older adults identified that the prototype frame’s novel lower handgrips safely supported them when transferring from sitting to standing or standing to sitting. The qualitative work identified that the frame’s new handgrips may be mostly useful to those living in the community rather than in care, and for them the new handgrips were perceived to facilitate independence and control. The frame was perceived to be an improvement on the status quo as it offers a new function that current standard frames do not offer. One approach would be to offer the novel frame design with/without the new handles, so that those not able to use them can still benefit from the other benefits the frame offers for walking, discussed in [21]. The significance of the research lies in the reported number of falls during transfer from sitting to standing and vice versa, with underlying causes reported including loss of support [31]. The proposed design is particularly timely considering the documented increases in frailty [32] and walking aid use [5] in our ageing population. Next, it will be important to trial the lower handgrips of the novel frame with novice frame users to further consolidate findings.

## Data Availability

The datasets generated and/or analysed during the current study are not publicly available due to the industry investment in the project and associated collaboration agreement which states that all IP generated remain with the company partner. However, data can be made available from the corresponding author on reasonable request and with permission of NRS Healthcare.
